# New thinking, innateness and inherited representation

**DOI:** 10.1098/rstb.2012.0125

**Published:** 2012-08-05

**Authors:** Nicholas Shea

**Affiliations:** Faculty of Philosophy, University of Oxford, 10 Merton Street, Oxford OX1 4JJ, UK

**Keywords:** evolutionary psychology, innateness, inherited representation, genetic information, cognitive evolution, cultural inheritance

## Abstract

The New Thinking contained in this volume rejects an Evolutionary Psychology that is committed to innate domain-specific psychological mechanisms: gene-based adaptations that are unlearnt, developmentally fixed and culturally universal. But the New Thinking does not simply deny the importance of innate psychological traits. The problem runs deeper: the concept of innateness is not suited to distinguishing between the New Thinking and Evolutionary Psychology. That points to a more serious problem with the concept of innateness as it is applied to human psychological phenotypes. This paper argues that the features of recent human evolution highlighted by the New Thinking imply that the concept of *inherited representation*, set out here, is a better tool for theorizing about human cognitive evolution.

## 1 Introduction

### (a) New Thinking versus Evolutionary Psychology

It is standardly assumed that innate psychological mechanisms should be the central focus of an evolutionary account of human cognition. One version of this approach is the ‘Evolutionary Psychology’ (capitalized) of Tooby and Cosmides with its series of special-purpose modules, each evolved to solve a particular problem in the Pleistocene [1]. Even outside high church Evolutionary Psychology, for example among those who point to more general-purpose evolved human faculties like the capacity for fine-grained visuomotor control, an evolutionary approach to human cognition is usually allied with a commitment to innate capacities.

A strong theme emerging from the papers in this volume puts pressure on the idea that there are innate psychological capacities at all. These are not blank slate empiricists or cultural determinists who deny the importance of evolution for explaining why we are as we are. If the New Thinking is on the right track, however, then innateness is not a useful concept for theorizing about recent human cognition.

Many papers in the volume emphasize domain-general adaptations: improved physical/causal understanding, increased visuomotor skill, and an extended childhood coupled with increased parental investment [2,3]. At a recent conference on the new thinking, Eva Jablonka argued that the hand is a much better metaphor than the Swiss-army knife for the special features of recent human cognition [4,5]. The hand is exquisitely complex and highly adapted, involving a suite of interdependent adaptive changes, but it is not adapted to any one particular task or outcome. It appears to have been selected instead for its facility as a generalist: to perform an open-ended range of tasks with great skill, where the concrete outcomes that contribute to fitness vary widely.

Two domain-general changes in hominin evolution are central here. The first is a marked increase in plasticity: in how quickly human individuals and social groups adapt to different ways of life in different ecological niches [6]. These developmentally plastic innovations depend for their development on rich support from the environment: information about particular variable features of the developmental environment, including resources and information derived from the ecological niche, from parents and from the wider culture [7].

The second change is a much greater reliance on culturally transmitted information. Among the adaptively-significant information on which development depends, some concerns facts that individuals could not learn for themselves in their own lifetime. In these circumstances individuals do not test and confirm for themselves that the developmental path they are adopting is adaptive; they rely on the information in their culture being a reasonably reliable guide to adaptive outcomes. So human behavioural development is extremely plastic, and some of that plasticity involves sensitivity to culturally transmitted information [7,8]. More strongly still, it is plausible that humans have adaptations for the cultural transmission of information, including through teaching and learning [9]. So, if cognition has evolved to be like the hand, it is a hand that was partly shaped by a culture of other hands, and which in turn plays a role in shaping other hands, being itself partly adapted to passing on the kind of cultural resources on which it depended on for its own development.

An evolutionary approach is often coupled with the thought that adaptive traits are innate. An innate trait is prototypically^1^ an adaptation that has evolved by gene-based natural selection, and is thereby coded in the genes. Its development does not depend on learning, and it would develop even if experience were impoverished in certain ways. Correlatively, it is relatively developmentally fixed in the face of environmental variations, and perhaps canalized against such variations. Indeed, on some views an innate trait is genetically determined from birth. And it is universal in the species, either in the sense that all members have it apart from non-typical cases in which there is an explanation of their exceptional status or, if there is typical variation in how the trait is manifested, then some shared basis for that variation is universal.

Evolutionary Psychology aims to account for the distinctive features of human life by appealing to special-purpose psychological capacities that have exactly those features: they are prototypically the result of gene-based natural selection, do not depend upon learning for their acquisition (and so admit of a poverty of the stimulus argument), are relatively developmentally fixed and hence culturally universal. This is in sharp contrast to the central features of the New Thinking identified earlier. So it might be thought that Evolutionary Psychology believes in, whereas the New Thinking denies, the importance of innate psychological capacities; and thus that the New Thinking makes less appeal to evolutionary considerations than high church Evolutionary Psychology.

That is a misdiagnosis: the New Thinking is just as motivated by evolutionary considerations. It relies heavily on selectionist explanation, phylogenetic methods and cross-species comparisons. It sees many domain-specific adaptations in human cognitive and social life. Some arise from gene-based selection, others from culturally based inheritance processes. Some are universal and others vary in different ecological and cultural contexts. Learning is involved in the acquisition of many of these traits, even when individuals are not able to learn about the adaptive significance of the trait for themselves. The New Thinking does not sit well either with accepting or denying that the relevant capacities are innate. That is because the concept of innateness is fundamentally unsuited to elucidating the contrast between the New Thinking and Evolutionary Psychology.

This paper will argue that the concept of *inherited representation* does a much better job of drawing the distinction. Evolutionary Psychology is committed to gene-based selection as the main source of the adaptively relevant information that is responsible for the adaptiveness of human psychological capacities; hence such capacities are unlearnt. The New Thinking emphasizes the complementary roles of gene-based and of culturally-based selection processes of various kinds for generating the adaptively relevant information on which human psychological capacities are based; hence the importance of interactions with the physical and social environment, and of learning, in developing those capacities.

### (b) Deeper troubles with innateness

Inutility is bad enough, but the troubles with the innateness concept run deeper. As philosophers, we want to know what property the concept of innateness refers to. That is, we want a theoretical reconstruction of the claim that a trait X is innate—we want to know what it is about a trait that determines whether the innateness claim is true or false. This exercise is not merely of philosophical interest. It is continuous with the scientific project of trying better to understand the properties that are appealed to in a scientific theory. To do so, we can observe the pattern of inferences in which a theoretical concept such as *innateness* is deployed, and seek to understand what the property is that underpins those inferences: that makes them defeasible but reasonably reliable ways of reaching true conclusions from true premises. The deeper problem with the concept of innateness is that there is no good account of what the property of being innate could be, so as to underpin the ways that the concept is used in relation to human psychological capacities.

The concept of innateness is used to make inferences between various properties like those mentioned earlier: developmental fixity, lack of learning and genetic coding—‘i-properties’ (adapting the terminology of Mameli & Bateson [17]). For instance: X is an adaptation → X is innate → X's development does not depend upon learning. That inference is highly unreliable because, as the New Thinking points out, many recent human adaptations depend crucially on learning. The problem is not just that the concept is sometimes used to make bad inferences. Rather, the problem is that there is no good proposal about how the concept could be used to make reliable inferences. What could the property of being innate amount to, such that the conclusions about human psychological capacities drawn using the concept should be reliable? If there were occasional exceptions, the concept would still be useful. If there were systematic exceptions, they could be guarded against in deploying the concept. But the concept is much more problematic if there is no property at all that would make inferences among the i-properties generally reliable in relation to human psychological traits.

The New Thinking puts pressure on the possibility of a satisfying theoretical reconstruction of the concept of innateness in two ways, one familiar, and the other less so. Its richly interactive picture of development undermines the tie, mediated by the innateness concept, between adaptation and developing without reliance on experience. There has been selection for a variety of clever behavioural phenotypes, such as the ability to process foodstuffs, or to make tools, or to cooperate with others in a hunt. Extended childhood and increased parental investment allow the development of many human phenotypes to be more richly interactive with the environment for a longer period. These changes in lifeways are themselves plausibly adaptations [19]. Evolution does not care about the devious and richly interactive route by which some clever behavioural phenotype has developed. It sees the phenotype that is the end point of development, and selection acts on that. Plausibly, many are adaptations. That developmental process happens to depend very heavily on learning and culture. When development is so richly interactive, depending upon all kinds of material and informational support from the structured ecological environment, and from other people, it is just a mistake to link being an adaptation with developing relatively independently of experience.

Furthermore, the emphasis on plasticity severs any potential link between adaptation and developmental fixity. Typical human innovations develop in a way that is open to environmental information, so as to fit in with variations in the environment. Even if such outcomes are robust against some forms of environmental variation, it is misleading to think of phenotypes whose development depends upon variable features of the environment as being relatively fixed or canalized against environmental variation. In short, in recent human evolution at least, there is no tie between being a phenotypic adaptation and development's unfolding in a certain way (i.e. being fixed and/or independent of experience).

The interactive nature of development has long been the basis of critiques of the innateness concept [20]. Standard counterexamples suggest that even universal adaptations need not be particularly well-insulated against environmental variation. For instance normal skin structure in humans depends on dietary vitamin C. Nevertheless, it was still argued that, for gene-based adaptations, development is so organized that genetic information plays a special role. Granted, environmental factors give essential causal support to the development of an adaptive trait, just as they did when it was selected, but the information that allows it to be adaptive derives from the genes, not from the environment. That is the proposal offered by Konrad Lorenz in response to critiques of the concept of innateness [21]. His proposed theoretical reconstruction of the innateness concept is that innate traits are those whose development depends on genetic information. That line is challenged by the second feature of the New Thinking identified earlier: the fact that much adaptively-significant information is culturally transmitted.

Although some forms of cultural evolution remain controversial, there are a variety of ways that broadly cultural processes accumulate adaptively-relevant information that is relied on for the development of adaptive phenotypes [22]. It has been argued that in humans gene–culture coevolution accelerated this process through positive feedback [6], both increasing the number of phenotypes that depend in part on gene-based adaptation, and magnifying the extent to which human cognitive development relies on culturally transmitted information. That undermines the possibility of even a rough dichotomy between individuals learning for themselves and relying on genetic information. Reliance on culturally transmitted adaptively-significant information forms an important third class: neither genetically transmitted and present at birth, nor just a matter of individuals learning for themselves about their environment—since cultural processes allow development to be sensitive to information beyond that available in the lifetime of an individual.

The other leading proposal for a theoretical reconstruction of the concept of innateness is that it just picks out the property of being unlearnt [14]. That would make sense of the way the innateness concept is used in relation to human psychological capacities if traits that depend on learning tend not to have the i-properties, i.e. tend not to be species typical, nor developmentally canalized, nor adaptations. The difficulty for that construal is that no way of making the concept of learning more precise does the job, as will be argued later. To preview: if learning is understood narrowly then many species-variable, developmentally plastic traits will count as unlearnt, hence innate. On the other hand, if learning is understood widely to include all the cases where an individual extracts information from the environment during development of a psychological trait, then many universal developmentally canalized traits, both adaptations and non-adaptations, will count as learnt, hence not innate. Neither way of delimiting what is to count as a learning process will make inferences among the i-properties generally reliable in respect of human psychological capacities.

In short, even if the concept of innateness works well enough in other biological domains, it is problematic with respect to the phenotypic traits that have arisen in recent hominin evolution. Nevertheless, the New Thinking is a thoroughly evolutionary approach. What replaces claims about innateness? This paper argues that the innateness concept should be replaced by the concept of genetic representation, and its generalization to epigenetic and cultural inheritance systems: inherited representation. Inherited representation is not a direct substitute for innateness, but allows us to articulate the way in which selection and adaptation are central to the New Thinking. It is suited to explaining aspects of phenotypic development that the abandonment of the concept of innateness would leave unexplained. Section 3 makes that case, and §2 lays the foundations by setting out an account of the semantic information carried by inheritance systems as it applies in other biological domains.

## 2 Genetic representation

### (a) Information generated by natural selection on genes

The first step is to see how genetic representation explains what is going on with innateness claims in other cases, outside human cognition. Then §3 argues that the concept goes particularly badly awry when applied to the results of recent human evolution.

The concept of innateness is applied on the basis of, and used to make inferences to, the various i-properties mentioned earlier. We have seen that the robustness of the connection between these properties and being a genetically-based adaptation has been seriously questioned. Nevertheless, evolution produces exquisitely adaptive phenotypes, and one feature of how they develop would be entirely mysterious but for the theory of natural selection.

Consider the close match between the bills of the purple-throated carib hummingbird *Eulampis jugularis* and the shape of the flowers on which they feed. Males develop bills that match the short straight flowers of *Heliconia caribeae*, and the smaller females develop longer curved bills that fit perfectly into the long curved flowers of *Heliconia bihai* [23]. The hummingbird somehow manages to develop a bill that will perfectly match a particular species of flower, but not as a result of causally interacting with the flower during development. The shape of the bill carries information about the shape of the flowers on which they feed. That information does not derive from interacting with the flower shape during development. So where did it come from? How does the developing hummingbird ‘know’ what shape of bill to produce, to match available nectar sources in its local ecology? There is evidence that the bills and flowers have coevolved, so the same question can be asked about the development of *Heliconia* flowers [24].

The same question is just as pressing for behavioural traits such as those we are considering here. How does the animal know how to behave without learning [25]? More carefully, how does the developing individual acquire a disposition for species-typical adaptive behaviour when the information that enables the behaviour to match the environment has not been derived from the environment during development? Of course we know that natural selection explains how adaptations arise, but it is less remarked that it also provides an answer to this informational question about development. The developing individual does not need information because there has been natural selection for a developmental process that arrives at the appropriate phenotype reasonably reliably. At some point a genetic variant or variants arose that biased development in the direction of a particular phenotype, which affected fitness and was selected. Neither at the stage of selection, nor subsequently, did development need to pick up on information about the environment. How, then, did the informational connection between adaptive phenotype and environmental feature arise? By virtue of selection—increasing the frequency of the variant which adaptively matched the environment. The developmental outcome of hummingbird bill development now carries information about flower shape by virtue of a history of selection in a population, increasing the frequency of matching variants.

### (b) Genes carry semantic information

This account of how genetic information plays a role in individual development can be made more precise using the philosophical concept of *genetic representation*. We can distinguish between the kind of information studied by information theory, based on correlations between one thing and another, and *semantic information*, in which a sign or representation supports a distinction between correctness and incorrectness, or being satisfied versus not. Correlational information is absolutely ubiquitous. Semantic information is much rarer in nature, but very familiar in ordinary life. It is the kind of content carried by sentences and thoughts.

Heritability at the time of selection means that at the time of selection genetic variants carry correlational information about phenotypic variants. And very many factors that make a causal impact on development, including the genome, carry correlational information about phenotypic outcomes—had the factor been different, the outcome would have been different. Most of these factors just carry correlational, not semantic information. The claim that there is genetic representation is the claim that genetic material also carries information of the stronger, semantic variety. Roughly the idea is that genes do not just happen to carry correlational information, but they are supposed to—in the unobjectionable naturalistic sense of ‘supposed to’ underpinned by evolutionary functions and natural selection.^2^

The case for genetic representation is founded on the fact that DNA does not just happen to be a means of transmitting phenotypes down the generations. There is good evidence that it has undergone selection for the way it performs that function [26,27]. That is to say, in addition to the evolutionary functions acquired by individual genes, the whole apparatus of DNA coding, translation, transcription and replication has the meta-level function of transmitting phenotypes down the generations. When a new genetic variant arises that correlates with a phenotypic difference, natural selection can act. Those correlations can be very long range, with the phenotypic variants being the result of a long and interactive process of development. Still, the result is that the selected genetic variant and its associated phenotypic variant increase in frequency, hence carry correlational information about the circumstances of selection. The DNA system is designed to transmit that phenotype to future generations (by transmitting the genetic variant with which it correlates). Now consider the zygotic DNA in an individual in a subsequent generation. For the system of DNA expression to perform its meta-function it should react to the selected gene by producing, by the same long interactive process of development, the phenotype for which it was selected.

Those facts mean that the genetic system falls within a simple model for characterizing the contents carried by low-level representing systems [28–30]. The elements are a ‘consumer’ system with the evolutionary function of responding to a variety of different representations by producing a variety of different outcomes, and some method by which representations are produced by which they correlate with external factors in such a way that the outputs of the consumer system are able to perform their evolutionary functions. A simple example is the honeybee nectar dance. ‘Consumer’ bees respond to the dance of an incoming worker by flying off a certain distance and direction in relation to the sun. That behaviour performs its evolutionary function on condition that there is nectar at that distance and in that direction. The behaviour of the consumer bees manages to fulfil that evolutionary function because incoming worker bees produce dances that correlate in a corresponding way with the location of nectar. The correlation produced by the incoming workers is an ‘exploitable relation’ [30], which outgoing consumer bees make use of, in the sense that the correlation is a reasonably reliable indicator that a condition on the behaviour they will perform being useful is in place.

In the genetic case the genes in the zygote are the representations and the whole long extended interactive processes of development are the consumer, eventuating in the phenotypic features exhibited by the organism. (DNA is also active within individual cells during development, but for present purposes that is a distraction, because it is not part of the reason why zygotic DNA has the representational content it does.) Because DNA has the exotic meta-function mentioned earlier, its job is to react to individual genes by producing, possibly at the end of a long and complex developmental process, the phenotypes for which they were selected. Development makes use of the correlation between genes and the environments in which they were selected as being a reasonably reliable indicator that the current environment is conducive to the phenotypes that result. If the environment has changed in relevant ways since the time of selection, that condition will no longer be in place, so the content carried by the genetic representation will be false. For example, if the selected phenotype depended upon the environment being warm, and it has since become cold, then the condition that the selected gene is representing—a warm environment—is absent. The genetic material then continues to represent (falsely) that the environment is warm and DNA expression and development, in accordance with its meta-function, faithfully produces a warm-appropriate phenotype as result. That phenotype is no longer good for the organism. This failure is explained by the fact that the content represented by the zygotic DNA is now false.

This account of genetic representation has been developed in detail elsewhere [31–34]. It is not simply a philosophers' gloss on well-known facts about natural selection. It has various explanatory benefits, for example when natural selection makes choices between relying on genetic or environmental information about some fitness-relevant factor [35,36]. For our purposes the key payoff is that it offers an explanation of the question we started with: how the developmental process in an individual manages to achieve an adaptive match to its environment without deriving from the developmental environment all the adaptively-relevant information that is actually carried by the adaptive phenotype. For example, an individual *E. jugularis* female ends up with a bill that so carefully matches the shape of the *H. bihai* flowers that it will feed from because the zygote contains genetic material that instructs the development of a long curved bill, and indicates that the environment is likely to contain flowers conducive to that shaped bill. That is why the individual does not need to interact with the shape of the flowers, or derive information about their shape in any other way from its own experience.

If DNA were the only inheritance system, then a developing individual would have access to only two sources of adaptively-significant information: genetic material, and the correlations that it could detect in the course of its own development. In fact the sources are much wider. Transgenerational epigenetic effects allow an individual to make use of information detected by its parents in their lifetime [37], as do parental effects mediated by other means. Some epigenetic effects may be transmitted with high fidelity down many generations, in which case they can accumulate information by selection on epigenetic variants, as we saw with genes, as mentioned earlier [38]. If some epigenetic system turns out to have been selected in part because of its ability to transmit phenotypes down many generations, then it too will have the earlier-mentioned meta-function. I call any mechanism with that meta-function an *inheritance system*. The selected phenotypes that are so-transmitted are *inherited representations* (*genetic representations* are one kind). Most importantly for our purposes, human evolution seems to have depended in part on one or more cultural inheritance systems. In that case, some of the information on which human phenotypic adaptations depend for their development derives neither from the genome, nor from the individual detecting correlational information available within its own individual experience, but from the developing individual reacting to information in its culture. Cultural processes of selection and transmission account for the way such cultural variants carry correlational information about adaptively-significant facts.

### (c) Genetic representation underpins some connections between i-properties

Genetic representation explains why the properties associated with the concept of innateness cluster together in some cases. In organisms with little or no culture there are two main sources of adaptively-relevant information. First, there is information that individuals can detect in their developmental environment in their lifetime. Second, there is the information conveyed by genetic representations (and, if they turn out to be empirically significant, by epigenetic representations that are carried along with the genome). A phenotype that develops in reliance on a genetic representation may adaptively match its environment in respects that the individual has not detected in its own lifetime. So there is a connection with poverty of the stimulus arguments. The trait can develop in the absence of certain kinds of information about the environmental feature it comes adaptively to match. For example, the female hummingbird *E. jugularis* develops its long curved bill even if deprived during its development of anything that provides information about the shape of *H. bihai* flowers.

That explains a link between genetic representation and one of the i-properties. There is also likely to be a certain amount of developmental fixity in a trait that develops in reliance on a genetic representation. To have been selected, the trait must have developed reasonably robustly across the kinds of environmental variations encountered when it was selected. To the extent that current environmental variations fall within that range, it is likely that the trait will arise in development despite such variations. If under continuing selection pressure, the trait may have even become developmentally canalized against such variations.

Adaptive polymorphisms have sometimes been thought to be an exception to this principle. If we adopt the right grain of analysis, it becomes clear that they are not. Evolution has selected for a developmental program that produces different outcomes in different environments, with some cue detected in development responsible for the fact that an individual ends up with the variant that adaptively matches its particular environment. That developmental disposition has a causal basis, which must itself develop. So it is that causal basis, rather than the various polymorphic outcomes of development, that is likely to be developmentally fixed, at least across the range of environments in which it evolved. The adaptive match of the eventual mature phenotype to its environment is partly due to genetic representation and partly due to adaptive reliance on environmental information. We will see later that such mixed models have particular importance in the human case.

Although a trait that develops in reliance on a genetic representation is likely to be fixed or canalized against the kinds of environmental variations present when it was selected, there is no reason to expect its development to be causally independent of the environment. Gene-based natural selection works on the basis of correlations between some genetic variant and some phenotypic outcome, correlations that are reasonably reliable at the time of selection. Those correlations can be very long range indeed. A genetic variant can bias the production of one phenotype over another by all sorts of devious means, and the pathway leading from zygotic DNA to phenotypic outcome can be intricate and interactive without limit. The informational contribution of a genetic representation is compatible with extremely rich causal contributions of the environment to the development of the trait.

Taking stock: recognizing the role of genetic representations in development explains why, in the absence of cultural inheritance systems, the following properties should co-occur better than chance: being a universal or species-typical adaptation, developing in a way that is invariant across some range of environmental variations, and there being an aspect of the adaptive information carried by the trait about its environment that is not the result of learning and about which a poverty of the stimulus argument could be made. These connections admit of exceptions, for a variety of reasons, but they co-occur often enough to make inferences between these properties go right better than by chance. So when a non-human trait is labelled ‘innate’ and taken to have these properties, the inferences it underpins will be reasonably reliable.

## 3 Inherited representation

### (a) Adaptation through cultural transmission

We cannot be so sanguine about innateness claims in the domain of human cognition. The rich interactivity of human behavioural development firmly severs any connection of gene-based adaptations with genetic determinism, or with their development being less causally dependent on the environment. That is the more familiar point about innateness that human evolution raises in a particularly pressing way. It is the New Thinking's conclusion that culturally transmitted information has been particularly important in recent human evolution that fatally undermines any utility of the innateness concept.

Sometimes when individuals get information from social sources they still learn for themselves. For example, with stimulus enhancement the individual learns for itself, through trial and error, how to perform some useful novel action on or with the stimulus. Nevertheless, with many of these social sources of information, a component of the adaptively-relevant information is being contributed by a cultural process. It consists of information the individual did not detect for itself. In some cases, it gives the individual access to a source of information that they could not detect for themselves.

Learning by ‘overimitation’ offers a clean example of this point. Both humans [39] and other primates [40] are capable of copying the means by which a demonstrated action is performed. Both can appreciate the difference between means to an intended goal and irrelevant actions [41–44] (cf. [45]). There is evidence that human children, unlike other apes, have a tendency to copy all the actions demonstrated in obtaining some desirable outcome, not just those that are causally necessary to obtaining the outcome. Chimpanzees, in contrast, cut out action steps that seem to them causally unnecessary [41,46].

This contrast is apparent in experiments using two versions of a mechanical box. In the transparent version the mechanism is visible so it is apparent when some actions performed by the demonstrator are causally irrelevant to achieving the goal of getting a reward out of the box. When the action sequence is demonstrated on an opaque box, it is not apparent that some of the action steps are unnecessary. Both chimpanzees and human children copy all the action steps performed on the opaque box in order to obtain the reward. With the transparent box, chimpanzees cut out the unnecessary action steps and go straight for the reward. Human children, in contrast, have a tendency still to copy all the demonstrated actions, even those that, by their own lights, are unnecessary to obtain the outcome. (That the children understand that some steps are unnecessary can be demonstrated in other experiments.)

There also seems to be a developmental trajectory. At the age of 12 and 18 months infants are focused on outcomes and tend not to copy specific demonstrated actions [47]. Three-year-olds are capable of copying specific actions [48] but less inclined to imitate unnecessary steps in the sequence. By the age of five years, children imitate obviously irrelevant actions in a wide range of conditions [49–52] (but cf. [53]). Overimitation appears to be even more pronounced in adults [54].

The human tendency to overimitate can seem paradoxical—would it not lead to the spread of useless or even detrimental behavioural variants?—until we realize that making individuals' tendency to imitate less sensitive to individual learning can increase its fidelity as an information transmission system [55]. The fidelity of cultural transmission can have a large impact on the power of cultural inheritance mechanisms [56]. If humans have a tendency blindly to imitate the demonstrated intentional actions even when, by their own lights, various of the actions are causally otiose, then this would allow the transmission of behaviours the utility of which is not apparent to any individual. So the tendency to overimitate may itself have evolved—genetically, or perhaps even culturally [9]—for the sake of transmitting behavioural phenotypes in a reasonably high-fidelity way down the generations.

For example, the connection between food preparation practices and nutritional outcomes may be so stochastic and variable that it is realistically impossible for a single individual to keep track of which practices have positive and negative impacts on fitness. But if lineages of individuals tend to adopt the same practices as their parents, then more beneficial variants will proliferate on average, because of their positive effects on the fitness of the individuals who have them. Similarly, the connection between action and outcome may be too long-term for an individual to detect it for themselves (cf. the link between smoking and cancer, which it took modern epidemiological statistics to detect).

Thus, given the right evolutionary conditions, the type of behavioural dispositions found in a population, which have been transmitted by overimitation, will carry information about past environments. Those that were beneficial to fitness in past environments will be found at higher frequency. To the extent that the current environment is relevantly similar to those past environments, the current pattern of behavioural dispositions carries adaptively-relevant information about features of the environment. That information has not been generated by individual learning, nor by any individual being able to detect the adaptively-relevant feature of the environment for themselves. Rather, it is selection over evolutionary time that has built up the information in the population. When a new individual adopts a behavioural disposition by overimitation from its parents, it is relying on adaptively-relevant information that has been generated by selection. But unlike genetic representation, the information carried in the overimitation-based cultural inheritance system (if there is one) is not present at birth.

That undermines a clean distinction between inherited information that is present at birth and acts as a constraint on the developmental trajectory (e.g. genetic representations), and adaptively-relevant information that individuals learn for themselves in their own lifetime. Culturally transmitted information is acquired by individuals in the course of their own lifetime, but individuals do not learn for themselves that it is adaptively-relevant; indeed, they may not be able to do so. On any reasonable view, the richly interactive process by which these traits develop counts as learning. So if innateness were simply equated with being unlearnt, as suggested earlier, these traits would not count as innate. That would overlook the fact that they share many properties with gene-based adaptations that are paradigmatically innate. Their development is likely to be relatively fixed in the face of the range of variation encountered in the developmental environments in which they were selected. Indeed, their development may be have become canalized against environmental variation by means of other culturally  or genetically inherited processes (gene–culture coevolution in the second case). And since the adaptively-relevant information on which they are based was generated by selection, their development will be susceptible to a poverty of the stimulus argument: the relevant information about an adaptively significant feature of the environment may not be detected or detectable in the course of individual development. Cultural selection gives rise to traits that would count as non-innate on the ‘learning’ definition, but for which the standard inferences drawn about non-innate traits will go awry.

Other forms of cultural transmission depend to a greater extent on individuals being able to learn for themselves [57]. For example, the most successful strategies in the social learning strategies tournament would try out observed behaviours for themselves and only add the behaviour to their repertoire if they themselves received a benefit for the behaviour [8]. That was a more efficient strategy than trying out a behaviour at random, because behaviours at high frequency in the social environment were more likely to be beneficial than a strategy picked at random. That is to say, the frequency of behavioural variants in the social environment carries information about what is adaptively useful. How is that information generated? In part through individual learning: high frequency behaviours are those that other individuals have tried out and found to be rewarding. But part of the information is generated by selection, because behavioural dispositions are inherited from parents and the payoffs obtained by an individual affect the number of offspring they have.

That mix is characteristic of the usefulness of culturally transmitted information. Some is information that others have learnt for themselves, so the imitator is short-cutting the need to learn something that they could, if necessary, have learnt from their environment. Other aspects of culturally transmitted information are generated in the transmission process itself, as a result of selection. In the latter sense individuals are doing more than ‘standing on the shoulders’ of the learning done by earlier individuals; cultural transmission is giving rise to adaptively relevant information that is more than the sum of the individual learning of the individuals who make up the culture [22].

### (b) A central role for culturally transmitted information

The argument that cultural transmission plays a central role in generating adaptively-relevant information faces two obstacles. The first is a tension between individuals learning for themselves, and cultural transmission building up information through natural selection. As noted earlier, the ability of individuals to learn for themselves and adopt the behaviour that seems best to them, from their own limited experience, introduces noise into the system for cultural transmission of behavioural phenotypes. As Godfrey-Smith argues [22], the more transmission depends on the intelligence of the individuals, the less ‘Darwinian’ the process will be. It will be less well-characterized by the models of quantitative genetics, for example.

However, Godfrey-Smith argues that this at most undermines the applicability of Darwinian micro-evolutionary models to the process. At the macro level, phylogenies of cultural practices can be highly illuminating even if the transmission process is nothing like high-fidelity individual-to-individual copying [58]. Also, at an intermediate level that Godfrey-Smith calls ‘meso-evolution’, a Darwinian approach can explain the origin of complex adaptive traits, even with intelligent transmission, since it explains the spread of adaptively beneficial phenotypes, whose high frequency in turn increases the chances of the next cumulative step of adaptive progress being discovered by an individual in the population. In short, the mix of individual learning and information produced by selection, identified above, is compatible with a broadly Darwinian approach to understanding how individuals manage to produce phenotypes that adaptively match their environment.

The second problem concerns the sharing of information. Developing individuals' use of culturally transmitted adaptively-relevant information found within their society is a form of information sharing. How can such information sharing practices be evolutionarily stable in the face of the fitness advantage that could be garnered by an individual in defecting—keeping a piece of useful information to themselves [2]? Natural selection runs off fitness differences; so if keeping information to oneself has costs, both to oneself and others, then the selfish strategy will be selected if the costs to others are higher than to oneself. For example, there are obvious advantages to sharing information about good hygiene. If others adopt a hygienic practice that I have discovered, that will on average reduce the overall risk of disease in the population, and so will indirectly reduce my own disease risk. But suppose I can get some benefit to myself from the hygienic practice, and suppose also that it is practical to keep it to myself. Then, although I would do better in absolute fitness if I were to share the practice, I will do better in relative fitness if I keep it to myself. I gain a relative fitness advantage since the risk of disease, although higher for all, falls more on others than it does on me. Why then share useful information?

Part of the answer may lie in the inherent difficulty of keeping some of these forms of information secret. Behaviours are observable, and a behavioural pattern that must be performed repeatedly is likely to be observed by those in the same social group. A restriction to vertical transmission of behavioural phenotypes would also help. If parents are only inclined to demonstrate useful behaviours to offspring and close kin, then they derive a direct fitness benefit from sharing useful information. Interestingly, there is evidence that overimitation is more common in ‘natural pedagogical’ contexts [59,60]. So far natural pedagogy has mostly been studied as a modifier of when children are disposed to learn. If it also restricts when adults are inclined to teach, then it could, in the evolutionary past, have constituted a system whereby lineages of behavioural phenotypes transmitted by overimitation roughly aligned with parent–offspring lineages, and hence with biological fitness.

A restriction to roughly vertical transmission will not cover anything like all of the cases. A more important part of the answer may lie in group selection (in the specific sense that between-group relative fitness benefits outweigh within-group relative fitness costs). If there is strong group selection then the fitness advantage of being in a group that shares information, vis-à-vis individuals in less informationally open social groups, may outweigh the relative fitness benefits to be garnered by selfishly keeping useful information to oneself. Although there is widespread scepticism about the importance of group selection in other species, it may well have been a particularly powerful influence in recent hominin evolution [61,62]. There is archaeological, palaeontological and anthropological evidence that humans lived in tight-knit social groups, perhaps extended kin groups in the first instance, with important variations between groups in culture, behavioural practices and even way of life. Powerful group selection would make a tendency to transmit adaptively relevant information throughout the social group less puzzling.

In sum, the following factors have probably played a role in recent human evolution: (i) high-fidelity transmission processes that closely model genetic micro-evolution; (ii) more diffuse ways that cultural transmission can build up adaptively-relevant information through selection; and (iii) cultural transmission as a means for disseminating and preserving useful information that individuals have learnt for themselves, allowing subsequent individual learning to ‘stand on their shoulders’. The relative importance of these factors remains an open empirical question.

### (c) No clustering of i-properties for human psychological phenotypes

These considerations mean that human development is likely to be particularly reliant on an interesting category of adaptively-significant information: information whose utility individuals are not wholly responsible for detecting for themselves, but information which, unlike more familiar forms of inherited representation (genetic, epigenetic), is picked up by the individual during her own lifetime. That decisively severs any connection between natural selection being responsible for the adaptive match between phenotype and environment, and the adaptively-relevant information being present at birth (in the zygote) and unlearnt. When the developing individual absorbs a culturally transmitted practice, or is steered by some culturally transmitted factor towards an adaptive phenotype, those are clearly cases of getting information from the environment. The information carried by these kinds of inherited representations is not information about which a poverty of the stimulus argument would succeed. It comes from (social) stimuli. The rough clustering of the i-properties in other species was underpinned by the fact that, if an individual did not learn the adaptively-relevant information for themselves, the information embodied in the adaptive match between a developmental outcome and the environment is very likely to have been generated by natural selection on genes, and to have been transmitted to this individual genetically, in factors that are present in the zygote and act so as to bias the complex interactive pathways of development towards the adaptive phenotype. Even that rough clustering will break down if the New Thinking is right about the importance of cultural transmission.

What about the universality or species-typicality of adaptive traits, that is presupposed by the concept of innateness? Recall that, when faced with adaptive polymorphisms in other species, I argued that there is plausibly a shared developmental program, designed to be guided by environmental information so as to produce different outcomes in different environments, each more adaptive in the environment in which it is produced. That does not carry over to the human case either. Or at least if it does, it is only in the most tenuous way. The New Thinking suggests that the ‘developmental program’ underpinning a huge swathe of polymorphic adaptive phenotypes is humans' highly adapted capacity for plasticity itself. That is a very domain-general ability. Of course, it too carries adaptively-relevant information—that the environment is likely to be very variable, that these are good ways to engage in learning—but what is absent is a more specific developmental program designed by evolution for a particular problem or set of problems. In short, extreme plasticity in some cases drives the nature of the potentially universal adaptation to the very highest, most general level. In other cases cultural inheritance may form the basis of universal, developmentally robust or even environmentally canalized human phenotypes.

In sum, an implication of the direction taken by the New Thinking in this collection is that the generalization that makes the i-properties cluster together roughly for non-human adaptations, giving the concept of innateness some utility there, is absent in the case of recent human behavioural adaptations. There is no particular reason to expect those human behavioural phenotypes that are plausibly adaptations to have the following features: to be due to natural selection on genes; to be unlearnt, or to develop such that aspects of the adaptively-relevant information on which they depend are not found in the developmental environment (such that a poverty of the stimulus argument could be made); to have a developmental course that is particularly invariant or canalized against environmental variations; or to be based on a universal developmental program, save the most domain-general capacities for plasticity, learning and cultural inheritance itself.

## 4. The new thinking articulated in terms of inherited representation

This paper has argued that recognizing the semantic information carried by genetic transmission down the generations—genetic representation—allows us to see why properties connected to the concept of innateness do tend to cluster together when applied to many biological adaptations. The utility of the innateness concept in those contexts makes it tempting to import it into our account of human evolution. High church Evolutionary Psychology does indeed claim that human psychological capacities are innate in a closely parallel sense: a suite of domain-specific adaptations produced by natural selection on genes, that are unlearnt, developmentally fixed and cross-culturally universal. The New Thinking points to evidence that argues against those claims. But the difference between the two viewpoints cannot be usefully encapsulated using the concept of innateness. The concept of inherited representation furnishes a clearer distinction. Evolutionary Psychology holds that genetic representation is by far the most important type of inherited representation responsible for human cognitive phenotypes. The New Thinking instead sees culturally transmitted inherited representations as an important source of the adaptively-relevant information present in human psychological phenotypes.

The characteristics that the New Thinking identifies as distinctive of human cognitive evolution also put pressure on the coherence of the concept of innateness, as it is applied to human psychology. The problem is not just that some of the inferences about innate traits that would go through in other biological domains fail in the case of human psychology. It is that there is no good proposal for a theoretical reconstruction of the concept of innateness that would explain why any significant portion of the inferences about human psychological traits standardly made using the concept of innateness should be reliable. If that is correct, then one important mistake of Evolutionary Psychology, which the New Thinking has transcended, is to make use of the concept of innateness. In contrast the New Thinking manages to be deeply evolutionary, while not being dependent on the concept of innateness at all.

That might be thought to leave a lacuna. How can the New Thinking account for the kinds of phenomena that innateness claims have been thought to explain? In this paper I have focused on one explanandum in particular: the adaptive match between phenotype and environment achieved as the end result of development. When that match is not wholly explained by the individual accessing adaptively-relevant information in its own experience, having detected its adaptive relevance itself, we are left with a puzzle: where did the information come from?

Inherited representation provides an answer, pointing to the multiple routes by which information generated by natural selection can constrain individual development towards an adaptive phenotypic outcome, so that aspects of the adaptively-relevant information carried by the phenotype need not derive from individual experience. Genetic and epigenetic mechanisms can play that role, giving rise to adaptively-relevant information that is present at birth; but we also saw three cultural routes to adaptive phenotypes, in none of which is the adaptively-relevant information present at birth, but which involve learning (albeit that individuals do not detect the adaptive significance of all the information for themselves).

Although cultural processes, learning and interactive development are central, the New Thinking is also thoroughly evolutionary, relying strongly on natural selection in explaining human psychology, unlike blank slate empiricism and the standard social science model. Phylogenetic methods and cross-species comparisons are relied on heavily, as part of the careful accumulation and assessment of all available evidence about the hominin and primate past. The importance that the New Thinking attaches to historical evidence shows just how serious it is about evolutionary explanation. However, using the concept of innateness to theorize about human evolution sits ill with the New Thinking. This paper argues that the supposed explanatory benefits of innateness claims can be salvaged, without residue, by identifying the significance for human evolution of various forms of inherited representation: genetic, epigenetic and cultural.

## EndNotes

1 This very condensed discussion is indebted to a very large literature on innateness, including: [10–18].

2 For convenience, I will talk in terms of genes carrying semantic information; in many cases the bearer of information will be something larger, like a chromosome or, in asexually reproducing species, the entire genome, even if the difference between the genomes on which selection is acting can be found at a single genetic locus.
